# On the Influence of Air in Shut Sacs

**Published:** 1814-09

**Authors:** 


### For the Medical and Physical Journal.

### On the Influence of Air in Shut Sacs; by Mr. ASHFORD.

HAVING, during an attendance lately at a celebrated northern university, heard much of the pernicious influence of air, when admitted into cavities from which it is naturally excluded, not only at the different societies of students by which that school is so much distinguished, but also from the chair of the professor, I became insensibly interested in the subject. I could not fail to perceive, that, in the discussions which occasionally arose, both the favourers and the opponents of that doctrine dealt much in general assertions; that on both sides there was a remarkable deficiency of proof; and that, whilst on the one hand it was rather assumed than inferred, on the other it was contradicted rather than disproved. I resolved, therefore, to employ some leisure hour in an investigation of the grounds on which that opinion had been advanced and maintained. In offering to your readers some of the reflections to which the inquiry has given rise, I am not influenced by an idea that they contain any thing novel or important; I have solely in view the calling of the attention of your readers to an interesting and neglected subject, wishing to receive rather than expecting to impart information.

Whatever may be the fate of this letter, I shall not regret having entered on the question to which it points. That time cannot be considered as misspent, in which we have learnt to appreciate more highly a cautious and distrustful spirit of induction; and, to the medical observer, no lesson surely can be more instructive than that which he derives from an observance of the weakness of which all conclusions partake that are drawn from too hasty a comparison of facts. Such a lesson I think a perusal of Dr. Monro's Observations* on the Influence of Air on "shut Sacs" is well calculated to afford. Whether the doctor's work was the first in which the subject was regularly examined, I have not had leisure to inquire. I may, however, I think, safely infer, that nothing very conclusive had been advanced by his predecessors, for as it appears to have been a great favourite with the doctor, he would not have failed to avail himself of every fact which could countenance and support it. Indeed there is some appearance of a claim to originality in the suggestion, for there is a most ominous silence of the hint having been ever before stated. Petit, moreover, is accused of not having

* Monro on the Bursæ Mucosæ.

The been aware of it, as constituting a principal argument in favour of returning the hernial sac unopened, which Petit was the first to perform, and the doctor the most forward to advise. Yet, whoever will read what that excellent surgeon has written on the subject, will find it expressly mentioned. An error of this kind is certainly quite pardonable: not so, however, the failing to acknowledge it when pointed out. It is this which has drawn from Mr. Lawrence a reproof not more severe than well deserved.

The doubts by which this question is obscured, evidently arise from the difficulty which we experience in determining how far the effects consequent on the exposure of a cavity are referable to the air admitted, or to the wound by which the admission is allowed. In tracing the inflammation which is apt to follow such wounds, Dr. Monro took the greatest advantage of this uncertainty which it is possible to conceive. To the influence of atmospheric air he attributes the more tedious cure of compound over simple fractures; the inflammation which follows a puncture of the dura mater to the admission of air to the brain. In no other way could he explain how the thrusting of a red-hot poker through the pericardium should prove fatal; and in three cases of tympanitis, in the first of which the colon was eroded by dysenteric ulcers, in the second two pins were sticking through the jejunum, and in the third the colon was fairly ruptured, he attributes the fatal event to the air effused. We may all probably recal to our minds some instance in which a cause has suffered as much from its advocates as from its opponents, and we are, I think, here presented with another example of the fact.

The support which Lewenhoek's discovery of the presence of animalculæ in the semen gave to that theory of generation which supposed the perfect rudiment of the fœtus to exist in the semen of the male, and the process of evolution and nutrition only to go on in the female, is well known. Lewenhoek at first certainly described what he saw, but intoxicated with applause, he soon began to substitute the dictates of fancy for the impressions of sense, and proceeded to advance assertions which staggered even the credulity of that age. Thus he alleged that in a single drop of semen he could distinguish *numerous animalculæ of different sexes*. This absurdity opened the eyes and cooled the ardour of physiologists, and thenceforth they began to reason more closely. In like manner, it might have been expected, that the latitude of conclusion in which Dr. Monro had indulged, would have led to an investigation of the whole of the evidence on which the doctrine was founded; yet we find Dr.

 Carmichael

Carmichael Smith echoing the same opinions without any apparent addition of facts.* Speaking of serous membranes, he says, "though not very sensible to the usual stimuli, they are affected in a singular manner by air, a stimulus which has little or no operation on other parts of the body endowed with the highest sensibility."

The rapidity with which inflammation is propagated in serous membranes from slight injuries, is certainly remarkable, and by superficial reasoners might well be considered as referable only to the general diffusion of a stimulus, such as air is supposed to be. I think it cannot be difficult to determine how far this is borne out by observation. If it can be shewn that the tendency to a spreading of inflammation takes place equally in cases of spontaneous inflammation (if I may be allowed the expression) as in those complicated with penetrating wounds, the conclusion against the doctrine is obvious, and I think inevitable. It cannot be necessary for me to refer to recorded instances for a proof of this property residing in them. Every practitioner's experience must have afforded him numerous examples of the fact. Is not the inflammatory blush extending over the surface of the intestines, a familiar consequence of enteritis? What is it that deters the surgeon from removing the hæmorrhoid by a ligature, but a dread of extensive peritoneal inflammation? Are we not all acquainted with the fatal consequences which have ensued from tying the whole spermatic cord after the operation of castration? To deny this property of serous membranes, is to prove oneself ignorant of the best-established fact in pathology. It is to peculiarity of structure then alone, that we are justified in referring the phenomenon in question. It is going out of our way to alledge the agency of air in the production of an effect to which it is clearly not essential. It is to multiply causes beyond the necessity of the case.

But the strongest arguments in favour of the innocency of air are drawn from an observance of the perfect safety with which an effusion of it is accompanied in cases of emphysema and tympanites. The experiments by Dr. Haighton† establish the same point. Taking advantage of the fact, that in some animals the communication of the tunica vaginalis with the abdominal cavity remains unclosed, he injected through it several cubic inches of air, by a puncture at the bottom of

* Medical Communications, vol. ii.

† See a paper on the Cæsarean operation in the "Medical Records and Researches."

the the scrotum, penetrating the tunica vaginalis—of a dog. Having repeated this experiment on several occasions, without any ill consequence, he says, "I do not hesitate to conclude that atmospheric air penetrating cavities is quite harmless." Now, I really should be glad to be informed on what grounds the contrary is still maintained. Have any subsequent experiments overturned Dr. Haighton's conclusions? Are they opposed by the result of any process of investigation equally free from objections, equally unequivocal and direct? Has their veracity been doubted? has their validity been disproved? or shall we never cease multiplying instances of the fact, that, as a theory may be advanced without proof, it may be *adopted without investigation, and persevered in, in spite of it*—Valent quantum valere debet.

*August* 8, 1814. ASHFORD.

